# A case report of feline mast cell tumour with intertumoral heterogeneity: Identification of secondary mutations c.998G>C and c.2383G>C in KIT after resistance to toceranib

**DOI:** 10.1002/vms3.70003

**Published:** 2024-08-23

**Authors:** Hiroyuki Tani, Tatsuro Hifumi, Keita Ito, Tomohide Kuramoto, Noriaki Miyoshi, Makoto Fujiki, Tomohiro Nakayama

**Affiliations:** ^1^ Laboratory of Veterinary Radiology Department of Veterinary Medicine College of Bioresource Sciences Nihon University Fujisawa Kanagawa Japan; ^2^ Laboratory of Veterinary Histopathology Joint Faculty of Veterinary Medicine Kagoshima University Kagoshima Japan; ^3^ Canine‐Lab. Inc. Koganei Tokyo Japan; ^4^ Kagoshima University Veterinary Teaching Hospital Joint Faculty of Veterinary Medicine Kagoshima University Kagoshima Japan; ^5^ Laboratory of Veterinary Surgery Joint Faculty of Veterinary Medicine Kagoshima University Kagoshima Japan

**Keywords:** acquired resistance, cat, heterogeneity, KIT mutation, mast cell tumour, resistance to toceranib

## Abstract

A 12‐year‐old male domestic cat with multiple subcutaneous mast cell tumours (MCTs) presented with a 2‐week history of pruritus and raw/bleeding skin from self‐trauma at Kagoshima University Veterinary Teaching Hospital. Polymerase chain reaction (PCR) and histopathological analyses revealed intertumoral heterogeneity among tumour locations based on the mutation status of *KIT*. In addition, the expression pattern of KIT was characterized. After failed treatment with vinblastine (2.0–2.2 mg/m^2^, intravenous administration, two doses in total) or nimustine (25 mg/m^2^, intravenous administration, two doses in total), toceranib (2.2–2.6 mg/kg, orally administered, every other day) was administered to treat recurrent MCTs harbouring the *KIT* exon eight internal tandem duplication mutation, achieving a complete response. However, toceranib resistance developed 2 months after treatment initiation. Subsequent PCR analysis was conducted to identify the mutational status of *KIT* in each MCT and to detect the presence of secondary mutations associated with the acquisition of toceranib resistance. Secondary *KIT* mutations (c.998G>C and c.2383G>C), which were not initially detected in tumour cells at diagnosis, were identified after the development of resistance to toceranib. This indicates that the tumour cells in feline MCTs in the same case have diverse characteristics. Our findings encourage further investigation into the development of therapeutic strategies for feline MCTs, particularly focusing on the heterogeneous nature of *KIT*/KIT and overcoming acquired resistance to toceranib.

## Graphical abstract

Key findings presented in the paper

To our knowledge, this is the first report of secondary KIT mutations (c.998G>C and c.2383G>C) in feline MCTs with intertumoral heterogeneity. Our findings encourage further investigation into the development of therapeutic strategies for feline mast cell tumours, particularly focusing on the heterogeneous nature of *KIT*/KIT and overcoming acquired resistance to toceranib.

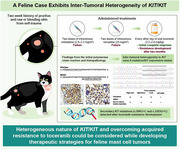

## 1 INTRODUCTION

Mast cell tumours (MCT) are common neoplasms that account for 21% of all feline skin tumours (Miller et al., 1991). Internal tandem duplication (ITD) of *KIT* exons 8 or 9 is the most common mutation identified in feline MCTs and is considered a driver mutation and therapeutic target (Isotani et al., 2010). Berger et al. (2018) reported the achievement of an objective response (complete or partial) to treatment with toceranib, a multikinase‐targeted drug, in 35 of 50 feline cases with an unknown mutation status for MCT (Berger et al., 2018). The targets of toceranib include the vascular endothelial growth factor receptor, platelet‐derived growth factor receptor, KIT, colony‐stimulating factor 1 receptor, and FMS‐like tyrosine kinase 3 (London et al., 2012; Tani et al., 2021). Although treatment with toceranib is well tolerated with favourable responses, it is discontinued in most feline cases because of toceranib resistance (Berger et al., 2018; Hasegawa et al., 2022). However, there are no reports on the mechanism of resistance in feline MCT. In the current study, we report a case of multiple subcutaneous MCTs with intertumoral heterogeneity of *KIT* exon 8 ITD mutations and the expression patterns of KIT. Moreover, we identified potential secondary somatic mutations in the tumour cells after resistance to toceranib.

## 2 CASE PRESENTATION

**FIGURE 1:**
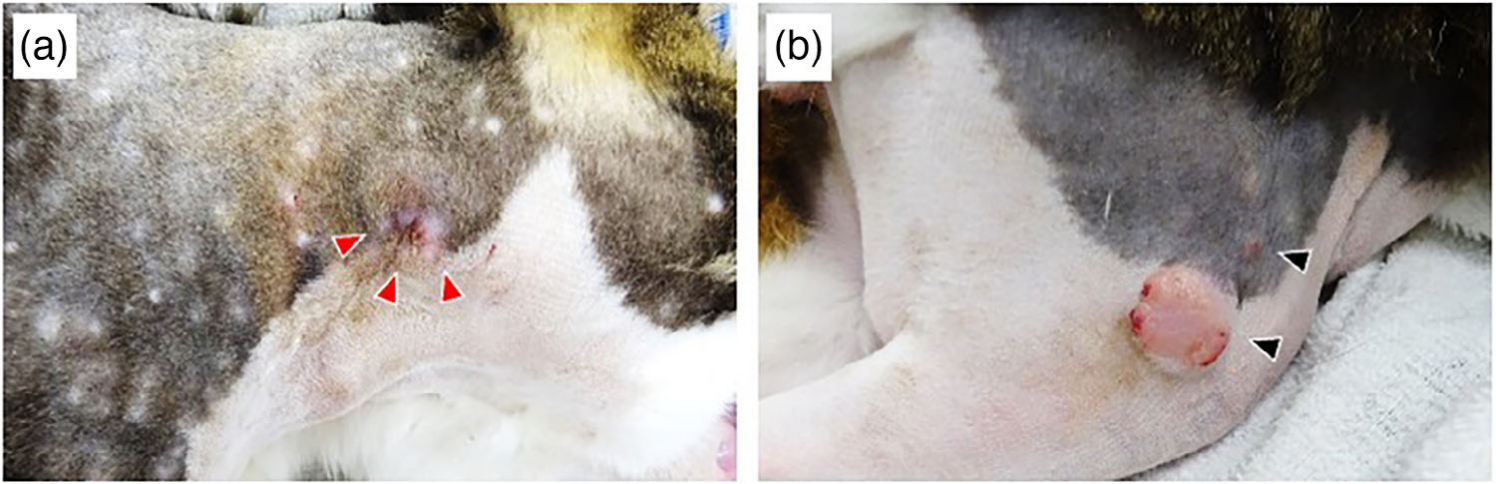
Clinical appearance of this case. Multiple subcutaneous masses on the right side of the neck (a) and femur (b). Bleeding is observed in the largest masses of both lesions: red arrowhead (a) and black arrowhead (b).

A 12‐year‐old male domestic cat weighing 4.9 kg presented to Kagoshima University Veterinary Teaching Hospital with a 2‐week history of pruritus and raw, bleeding skin from self‐trauma. Multiple subcutaneous masses were observed on the head, neck, trunk, and femur (Figure 1). Bleeding was observed in the masses on the right side of the neck and femur.

## 3 DIFFERENTIAL DIAGNOSIS

On the basis of the characteristic clinical signs, the most likely differential diagnosis was MCT. Other possible diagnoses, including cutaneous lymphoma, other benign or malignant tumours, bacterial or mycobacterial infections, and autoimmune diseases, were considered and ruled out.

## 4 DIAGNOSIS

Fine‐needle aspiration (FNA) was performed for the masses in the right neck, left cheek, right femur, trunk, and right mandibular lymph nodes. No abnormalities were observed in the blood examination or two‐view thoracic radiography. Abdominal ultrasonography revealed a slightly enlarged spleen, which was subsequently subjected to ultrasound‐guided FNA. These clinical examinations revealed subcutaneous MCTs with metastases to the spleen and right mandibular lymph nodes. MCTs were suspected to be the cause of the clinical symptoms. Six lesions of disease, the spleen and five subcutaneous masses located in the right neck, left cheek, trunk, and right femur, were selected for debulking surgery under general anaesthesia. The primary goals of surgery were to manage clinical symptoms and to establish a definitive diagnosis. These samples were utilized for analysing KIT mutations and histology.

**FIGURE 2:**
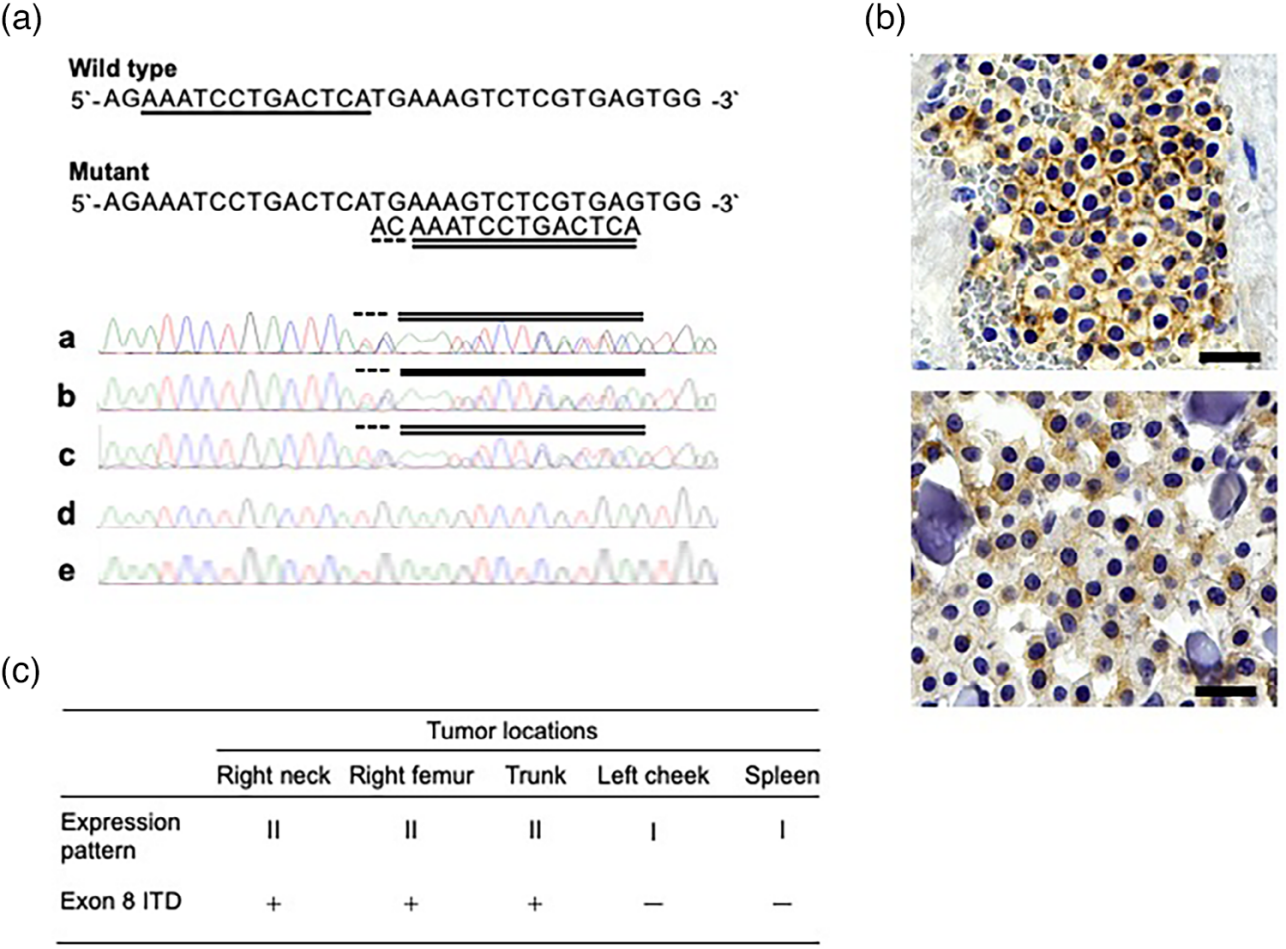
Identification of intertumoral heterogeneity in mast cell tumour (MCT). (A) Internal tandem duplication (ITD) of 13 nucleotides (underlined, wild type; double underlined, and mutant) and insertion of two nucleotides (broken line) were identified in genomic deoxyribonucleic acids extracted from MCTs. ITD were detected in MCTs located in the right neck (a), right femur (b), and trunk (c), but not in the spleen (d) or left cheek (e). (B) Immunohistochemistry for KIT expression and mutation status. Membranous (pattern I; upper panel) and focal/stippled cytoplasmic (pattern II; lower panel) expression patterns were observed in splenic and trunk MCTs, respectively. (C) Tumour location, KIT expression pattern, and ITD mutation status in exon 8. Bar, 20 mm.

Polymerase chain reaction (PCR) analysis of *KIT* mutations was performed using the CFX Connect Real‐Time PCR Detection System (Bio‐Rad Laboratories Inc.) and TB Green Premix Ex Taq (Tli RNase H Plus; TaKaRa‐Bio Inc.) at a commercial laboratory. Genomic deoxyribonucleic acids were extracted from paraffin‐embedded samples using phenol–chloroform and ethanol precipitation methods. Intronic primer pairs, designed on the basis of a previous report (Isotani et al., 2010), were used to amplify the entire *KIT* exonic region and perform direct sequencing (Table S1). The amplification products of genomic DNA were directly sequenced. The normal feline *KIT* complementary DNA nucleotide sequence (GenBank accession number NM_001009837.3) was used as a reference to identify mutations and to determine the number of nucleotides and amino acid sequences. Variations were observed in the presence or absence of ITD mutations in *KIT* exon 8 among the tumours (Figure 2A). The mutation was identified in the tumour cells present in the masses located on the right neck, trunk, and right femur, but not in those of the left cheek or spleen.

Each mass was fixed in 10% neutral‐buffered formalin, embedded in paraffin, and sectioned into 3 µm slices, which were then stained with haematoxylin and eosin and toluidine blue (pH 7.0). Immunohistochemistry was performed using the polymer method with a Histofine Simple Stain MAX PO (Multi) Kit (Nichirei Biosciences). Anti‐c‐kit/CD117 rabbit polyclonal antibody (1:400; catalogue no. A4502; Dako) was used as the primary antibody. Endogenous peroxidase activity was inactivated using 3% H_2_O_2_–methanol. Antigens were retrieved using autoclave heating (121°C for 15 min) in a 10‐fold diluted Antigen Retrieval Solution (pH 9.0) (catalogue no. 415211; Nichirei Biosciences). The detected antigens were visualized with 3,3′‐diaminobenzidine tetrahydrochloride reagent (Nichirei Biosciences). The sections were counterstained with Mayer's haematoxylin.

Histopathological analysis revealed that the five subcutaneous masses displayed similar characteristics, consisting of nodular, multifocal, or infiltrative proliferation of MCT cells originating from the dermis and subcutaneous tissue with invasion into the surrounding tissue. Mitosis was observed to a small extent (<1/10 of high‐power fields). Toluidine blue staining showed fine metachromatic granules in the cytoplasm of the neoplastic cells. The surgical margins of the subcutaneous MCTs were incomplete, and similar tumour cells with clustered forms were observed in the red spleen cord. These histopathological findings supported the diagnosis of subcutaneous MCTs with splenic infiltration.

The immunohistochemical results for KIT expression are presented in Figure 2B. KIT expression pattern was evaluated on the basis of a previous report (pattern I: membranous expression; pattern II: cytoplasmic expression; pattern III: diffuse cytoplasmic expression) (Sabattini et al., 2013). KIT expression was detected in the MCT cells of all masses. Pattern II expression was observed in the right neck, right femur, and trunk, whereas pattern I was detected in the left cheek and spleen. No Pattern III KIT expression was observed. Pattern II KIT expression was observed in tumours harbouring KIT mutations, whereas pattern I expression was observed in tumours harbouring wild‐type KIT (Figure 1c).

Day 1 was designated as the day of diagnosis. Treatment response was evaluated according to the Response Evaluation Criteria for Solid tumours in Dogs (Volume 1.0) (Nguyen et al., 2015), and adverse events were assessed according to the Veterinary Cooperative Oncology Group‐Common Terminology Criteria for Adverse Events (Volume 2.0) (LeBlanc et al., 2021).

## 5 TREATMENT

Treatment courses are listed in Table 1. One week after the diagnosis, multiple subcutaneous nodules were identified on the trunk (Day 7). Treatment with prednisolone (Predonine tablets; 2.0 mg/kg for 2 weeks and tapered to 1.5 mg/kg orally, once a day; Shionogi & Co., Ltd.) and diphenhydramine (Polaramine; 2 mg/head, orally, twice a day; TAKATA Pharmaceutical Co., Ltd.) was subsequently initiated. Prednisolone was discontinued on Day 42 due to diabetes; however, diphenhydramine was administered throughout the treatment period. On Day 66, a relapse of clinical symptoms (pruritus of the trunk) was observed, and tumour aggravation was suspected. Although toceranib could be considered an effective drug for MCT harbouring KIT mutations, vinblastine (Exal 10 mg; 2.0–2.2 mg/m^2^, intravenously bolus administration via vena cephalica; Nippon Kayaku Co., Ltd.) was selected as the initial cytotoxic chemotherapeutic agent for treatment due to the intertumoral heterogeneity of the mutation pattern. Vinblastine dosing was initially planned on a 12‐week protocol (4 weekly administrations, followed by four administrations every 2 weeks); however, it was performed every 2 weeks at the request of the client. On Day 78, disease progression was diagnosed on the basis of the appearance of multiple new lesions on the trunk and worsening of clinical symptoms. Side effects of vinblastine at the dose of 2.2 mg/m^2^, including grade I gastrointestinal symptoms (inappetence and vomiting), were also observed. On Day 91, the chemotherapeutic agent was changed to nimustine, a nitrosourea drug (nidran; 25 mg/m^2^, every 3 weeks intravenously bolus administration via the vena cephalica; Daiichi Sankyo Co., Ltd.). Regrowth of the tumour in the right neck and appearance of a new disease in the trunk were observed on Day 128. No adverse effects were observed following nimustine treatment.

**TABLE 1:** Treatment of 12‐year‐old male domestic cat with multiple subcutaneous mast cell tumours.

			Days								
			7	66	78	91	107	128	149	177	190
Antitumoral drugs (dosage)			Prednisolone (1.5–2.0 mg/kg, once a daily, orally)	Vinblastine (2.0 mg/m^2^, intravenously)	Vinblastine (2.2 mg/m^2^, intravenously)	Nimustine (25 mg/m^2^, intravenously)	Nimustine (25 mg/m^2^, intravenously)	Toceranib (2.6 mg/kg, EOD, orally)	Toceranib (2.2 mg/kg, EOD, orally)	Toceranib (2.3 mg/kg, EOD, orally)	Non
Treatment response	Over all		N.E.	PD	PD	PD	SD	PD	CR	CR	PD
	Tumour location (KIT mutation/ Expression pattern of KIT)	Right neck (exon 8 ITD/II)	Non	Non	Non	Relapse	SD	Regrowth	Non	Non	Relapse
		Right femur (exon 8 ITD/II)	Non	Non	Non	Non	Non	Non	Non	Non	Non
		Trunk (exon 8 ITD/II)	Multiple nodules	SD	Appearance of new disease	Appearance of new disease	SD	Appearance of new disease	Non	Non	Non
		Left cheek (WT/I)	Non	Non	Non	Non	Non	Non	Non	Non	Non
		Spleen (WT/I)	Non	Non	Non	Non	Non	Non	Non	Non	Non
	Clinical symptoms		Non	Relapse of pruritus	Pruritus and raw/bleeding skin from self‐trauma	Worsening of pruritus	Improvement of pruritus	Improvement of pruritus	Disappeared	Non	Non

Abbreviations: CR, complete response; EOD, each other day; ITD, internal tandem duplication; PD, progressive disease; PR, partial response; SD, stable disease; WT, wild type.

Treatment with toceranib phosphate (Palladia 10 mg tablets; 2.6 mg/kg, orally, every other day; Zoetis) was initiated on Day 128, leading to complete resolution of the gross disease and clinical symptoms. Toceranib treatment was well tolerated with no adverse effects. However, on Day 190, local recurrence of the MCT in the right neck was identified using FNA, and a relapse of clinical symptoms was noted.

Although unclear in feline MCT, a secondary mutation in KIT has been reported as one of the mechanisms underlying toceranib resistance in canine MCT (Kurita et al., 2019). To verify this, additional PCR analyses of the KIT exons 6, 8, 9, 11, 13, and 17 were performed (Supporting Information and Table 1). The PCR results are presented in Table 2. In addition to primary KIT exon 8 ITD, two novel point mutations, c.998G>C [p.E330Q] in exon 6 and c.2383G>C [p.A795R] in exon 17, were identified in recurrent MCT cells (Figures [Link], [Link]). These point mutations were detected as heterozygous and were considered missense mutations involving amino acid substitutions. Furthermore, these mutations were not detected in MCT cells from other locations. A silent mutation (c.1380G>A) in exon 9 was observed in the left cheek tumour. No mutations were identified in exons 11 and 13 of the MCT cells at any location.

**TABLE 2:** KIT mutation status of a 12‐year‐old male domestic cat with multiple subcutaneous mast cell tumours.

		Exon					
Mast cell tumour		6	8	9	11	13	17
Right neck	Primary	WT	c.1244_1256dup, c.1256_1257insAC	WT	WT	WT	WT
	TOC‐resistant	c.998G>C [p.E330Q]	c.1244_1256dup, c.1256_1257insAC	WT	WT	WT	c.2383G>C [p.A795R]
Right femur		WT	c.1244_1256dup, c.1256_1257insAC	WT	WT	WT	WT
Trunk		WT	c.1244_1256dup, c.1256_1257insAC	WT	WT	WT	WT
Left cheek		WT	WT	c.1380G>A [silent]	WT	WT	WT
Spleen		WT	WT	WT	WT	WT	WT

Abbreviations: TOC, toceranib; WT, wild type.

In the present case, we identified novel somatic missense mutations in KIT in a case of feline MCT with intertumoral heterogeneity after developing resistance to toceranib treatment. To our knowledge, this is the first report of secondary KIT mutations (c.998G>C and c.2383G>C) in feline MCTs. These mutations, newly identified during the clinical course, may be associated with toceranib resistance. In this case, the MCTs exhibited a range of mutation statuses and expression patterns at the time of diagnosis. This suggests that tumours consist of cells with different characteristics, indicating heterogeneity within or between tumours. This suggests the presence of a small number of treatment‐resistant cells prior to treatment. Furthermore, subsequent treatment with antitumour drugs may have favoured selective growth of these cells.

## 6 DISCUSSION

This case exhibited varying trends in the status of *KIT* mutations, KIT expression patterns, and responsiveness to therapeutic agents depending on the tumour location. The presence of tumour subcloned with different phenotypic and molecular characteristics that coexist within a tumour is referred to as intra‐tumour heterogeneity, whereas further differences between individual tumour types within the same patient are termed intertumoral heterogeneity (Burrell et al., 2013; Grzywa et al., 2017). Heterogeneity is also present in most solid tumours and haematological malignancies in humans, whereas genetic heterogeneity differs between primary and metastatic lesions (Grzywa et al., 2017). Subclonal populations of tumour cells with genomic instability can generate diverse phenotypic variations, including somatic point mutations. Some anticancer therapies may induce this subclonal diversity within tumours (Burrell et al., 2013).

Subclonal diversity is thought to be the mechanism underlying treatment resistance. In human acute myeloid leukaemia, anticancer therapy can induce the expansion of resistant subclones or secondary treatment‐resistant mutations (Ding et al., 2012). Moreover, heterogeneity is considered a prognostic factor in various human cancers, affecting the treatment response or the acquisition of resistance to targeted molecular therapies (Brady et al., 2017; Marusyk et al., 2020). The intra‐tumour heterogeneity of *KIT* in single‐site MCTs and the presence of multiple subclones have been previously reported in feline MCTs (Hasegawa et al., 2022). Differences in the treatment response to chemotherapeutic agents at the tumour site and in resistance to toceranib in this case may indicate the presence of subclones within or between tumours. In addition, in the present case, the differences between KIT expression patterns and *KIT* mutation status appeared to be linked to the tumour location (Figure 1c). Sabattini et al. (2013) reported that six of nine patients with multiple MCTs had different *KIT* mutation statuses and KIT expression patterns. Although no definitive conclusions have been drawn regarding the association between mutations and expression patterns in feline MCTs (Sabattini et al., 2013), Webster et al. (2006) reported a significant relationship in canine MCTs (Webster et al., 2006).

Reactivation of KIT by secondary mutations in exons 13 or 14 (ATP‐binding pocket) and 17 or 18 (activation loop) is a common resistance mechanism observed in 90% of patients with imatinib‐resistant human gastrointestinal stromal tumours (GIST). Intra‐tumour heterogeneity and imatinib‐resistant subclones may underlie this resistance mechanism (Serrano et al., 2019). In canine MCTs, resistance to targeted therapy caused by secondary mutations has been reported in the ATP‐binding pocket and the activation loop of KIT (Nakano et al., 2017; Gentilini., et al. 2020), the frequency of secondary mutations in cases of resistance to molecular therapies targeting KIT in canine or feline malignancies has not yet been reported.

In this case, two novel missense mutations, c.998G>C [p.E330Q] in exon 6 and c.2383G>C [p.A795R] in exon 17, were identified in the toceranib‐resistant MCT cells. *KIT* exon 6 encodes the fourth immunoglobulin‐like domain of KIT, whereas the fourth or fifth immunoglobulin‐like domain has been reported to stabilize stem cell factor‐binding extracellular domains in the human KIT homodimer (Blechman et al., 1995; Yuzawa et al., 2007). Therefore, although mutations in exon 6 may affect KIT activation, the nature of this mutation in canine or feline MCTs remains unclear. Mutations in exon 17, which encodes the activation loop, cause hyperphosphorylation of KIT under toceranib exposure in canine MCTs (Kurita et al., 2019) and are related to the acquisition of toceranib resistance. In the primary right neck tumour, a KIT mutation was identified in exon 8 but not in exons 6 or 17. These findings suggest that one or both novel secondary KIT mutations are associated with toceranib resistance. On the basis of previous reports on canine MCTs and human GIST, the secondary mutations found in this case were likely to be involved in the development of resistance to toceranib. Furthermore, as there have been no previous reports of secondary mutations in exon 6, this discovery may offer new insights into cell proliferation or drug resistance in feline MCTs. One limitation of this study is that no data support an association between secondary KIT mutations and toceranib resistance using either cell lines or recombinant feline KIT proteins. Additionally, direct sequencing of whole feline KIT exons was not performed; therefore, other unidentified secondary mutations may exist.

In the present case, multiple subcutaneous MCTs with splenic involvement were observed. Although FNA is commonly used for the clinical staging of feline MCTs with multiple nodules and splenic involvement testing, the mutational status of KIT for each mass site is not typical during the clinical course. Sabattini et al. (2013) highlighted that the intertumoral heterogeneity of *KIT* could limit the use of tyrosine kinase inhibitors in the treatment of feline MCTs. Toceranib is a small‐molecule‐targeted drug that acts on multiple tyrosine kinases, including KIT (Thamm et al., 2020). In this case, toceranib was not selected as a primary or secondary therapy because of the presence of multiple nodules with heterogeneous *KIT* mutations. As imatinib, a tyrosine kinase inhibitor, exerts anti‐tumour effects on feline MCTs harbouring *KIT* exon 8 or 9 ITD via the inhibition of phosphorylation (Isotani et al., 2010), the treatment response to toceranib in this case was assessed using a similar mechanism. Although the anti‐tumour effects of toceranib are comparable to those of vinblastine in canine MCTs with or without KIT mutations (Thamm et al., 2020), the relationship between the therapeutic response to toceranib and *KIT* mutation status in feline MCT remains unclear (Berger et al., 2018; Hasegawa et al., 2022). The diversity of KIT mutations and expression patterns observed in this case suggests that feline MCTs exhibit genetic variability. Comprehensive studies, including prospective studies, are necessary to assess whether the heterogeneity of feline MCTs influences the effectiveness of anti‐tumour therapy. Thorough genetic mutation testing may be necessary at the time of feline MCT diagnosis to select therapeutic drugs.

## 7 CONCLUSION

In this case, a 12‐year‐old male domestic cat with multiple subcutaneous MCTs exhibited marked differences in either the mutation status or expression pattern of *KIT*/KIT, which was dependent on the tumour location. No standardized initial treatment protocol has been reported for feline MCTs that exhibit intertumoral heterogeneity, as in this case, and future prospective studies are warranted. Moreover, two secondary KIT mutations in exons 6 and 17, which were identified in relapsed MCT cells, were considered to be related to the acquisition of resistance to toceranib. Although we could not perform an in vitro analysis, these mutations could potentially induce reactivation of KIT phosphorylation when exposed to toceranib. This case report highlights the importance of further research on treatment strategies for intertumoral heterogeneity and overcoming toceranib resistance in feline MCTs.

## AUTHOR CONTRIBUTIONS

Most of the clinical work was performed by Hiroyuki Tani in collaboration with Tomohide Kuramoto and Makoto Fujiki. All authors contributed to the conception and design of this study. Hiroyuki Tani, Tomohide Kuramoto, Tatsuro Hifumi, Keita Ito, Noriaki Miyoshi, Makoto Fujiki, and Tomohiro Nakayama prepared the materials, collected and analysed the data. Hiroyuki Tani supervised the study. Hiroyuki Tani wrote the first draft of the manuscript, and all authors commented on previous versions of the manuscript. All the authors have read and approved the final version of the manuscript.

## CONFLICT OF INTEREST STATEMENT

The authors declare no conflicts of interest.

## FUNDING INFORMATION

This study was partially supported by a Grant‐in‐Aid for Scientific Research (No. JP21K14991) from the Japan Society for the Promotion of Science.

## ETHICS STATEMENT

The authors confirm that the ethical policies of the journal, as noted on the author guidelines page, have been adhered to. No ethical approval was required because this was a case report.

### PEER REVIEW

The peer review history for this article is available at https://publons.com/publon/10.1002/vms3.70003.

## Supporting information

Supporting Information

Supporting Information

Supporting Information

Supporting Information

## Data Availability

The datasets used and/or analysed in the current study are available from the corresponding author upon reasonable request.

## References

[vms370003-bib-0001] Berger, E. P. , Johannes, C. M. , Post, G. S. , Rothchild, G. , Shiu, K. B. , Wetzel, S. , & Fox, L. E. (2018). Retrospective evaluation of toceranib phosphate (Palladia) use in cats with mast cell neoplasia. Journal of Feline Medicine and Surgery, 20(2), 95–102. 10.1177/1098612X17695898 29172873 PMC11129263

[vms370003-bib-0002] Blechman, J. M. , Lev, S. , Barg, J. , Eisenstein, M. , Vaks, B. , Vogel, Z. , Givol, D. , & Yarden, Y. (1995). The fourth immunoglobulin domain of the stem cell factor receptor couples ligand binding to signal transduction. Cell, 80(1), 103–113. 10.1016/0092-8674(95)90455-7 7529140

[vms370003-bib-0003] Brady, S. W. , McQuerry, J. A. , Qiao, Y. , Piccolo, S. R. , Shrestha, G. , Jenkins, D. F. , Layer, R. M. , Pedersen, B. S. , Miller, R. H. , Esch, A. , Selitsky, S. R. , Parker, J. S. , Anderson, L. A. , Dalley, B. K. , Factor, R. E. , Reddy, C. B. , Boltax, J. P. , Li, D. Y. , Moos, P. J. , … Bild, A. H. (2017). Combating subclonal evolution of resistant cancer phenotypes. Nature Communications, 8(1), 1231. 10.1038/s41467-017-01174-3 PMC5666005 29093439

[vms370003-bib-0004] Burrell, R. A. , McGranahan, N. , Bartek, J. , & Swanton, C. (2013). The causes and consequences of genetic heterogeneity in cancer evolution. Nature, 501(7467), 338–345. 10.1038/nature12625 24048066

[vms370003-bib-0005] Ding, L. , Ley, T. J. , Larson, D. E. , Miller, C. A. , Koboldt, D. C. , Welch, J. S. , Ritchey, J. K. , Young, M. A. , Lamprecht, T. , McLellan, M. D. , McMichael, J. F. , Wallis, J. W. , Lu, C. , Shen, D. , Harris, C. C. , Dooling, D. J. , Fulton, R. S. , Fulton, L. L. , Chen, K. , … DiPersio, J. F. (2012). Clonal evolution in relapsed acute myeloid leukaemia revealed by whole‐genome sequencing. Nature, 481(7382), 506–510. 10.1038/nature10738 22237025 PMC3267864

[vms370003-bib-0006] Gentilini, F. , Turba, M. E. , Dally, C. , Takanosu, M. , Kurita, S. , & Bonkobara, M. (2020). The secondary KIT mutation p.Ala510Val in a cutaneous mast cell tumour carrying the activating mutation p.Asn508Ile confers resistance to masitinib in dogs. BMC Veterinary Research, 16(1), 64. 10.1186/s12917-020-02284-9 32075643 PMC7029481

[vms370003-bib-0007] Grzywa, T. M. , Paskal, W. , & Włodarski, P. K. (2017). Intratumor and intertumor heterogeneity in melanoma. Translational Oncology, 10(6), 956–975. 10.1016/j.tranon.2017.09.007 29078205 PMC5671412

[vms370003-bib-0008] Hasegawa, Y. , Shosu, K. , Tsuji, K. , Shimoyama, Y. , Miyama, T. S. , Baba, K. , Okuda, M. , Itamoto, K. , Igase, M. , & Mizuno, T. (2022). Intratumoral heterogeneity of c‐KIT mutations in a feline splenic mast cell tumor and their functional effects on cell proliferation. Scientific Reports, 12(1), 15791. 10.1038/s41598-022-19089-5 36138037 PMC9499958

[vms370003-bib-0009] Isotani, M. , Yamada, O. , Lachowicz, J. L. , Tamura, K. , Yagihara, H. , Fujino, Y. , Ono, K. , Washizu, T. , & Bonkobara, M. (2010). Mutations in the fifth immunoglobulin‐like domain of kit are common and potentially sensitive to imatinib mesylate in feline mast cell tumours. British Journal of Haematology, 148(1), 144–153. 10.1111/j.1365-2141.2009.07926.x 19804453

[vms370003-bib-0010] Kurita, S. , Miyamoto, R. , Tani, H. , Kobayashi, M. , Sasaki, T. , Tamura, K. , & Bonkobara, M. (2019). Genetic alterations of KIT during clonal expansion and subsequent acquisition of resistance to toceranib in a canine mast cell tumor cell line. Journal of Veterinary Pharmacology and Therapeutics, 42(6), 673–681. 10.1111/jvp.12816 31553064

[vms370003-bib-0011] LeBlanc, A. K. , Atherton, M. , Bentley, R. T. , Boudreau, C. E. , Burton, J. H. , Curran, K. M. , Dow, S. , Giuffrida, M. A. , Kellihan, H. B. , Mason, N. J. , Oblak, M. , Selmic, L. E. , Selting, K. A. , Singh, A. , Tjostheim, S. , Vail, D. M. , Weishaar, K. M. , Berger, E. P. , Rossmeisl, J. H. , & Mazcko, C. (2021). Veterinary cooperative oncology group‐common terminology criteria for adverse events (VCOG‐CTCAE v2) following investigational therapy in dogs and cats. Veterinary and Comparative Oncology, 19(2), 311–352. 10.1111/vco.12677 33427378 PMC8248125

[vms370003-bib-0012] London, C. , Mathie, T. , Stingle, N. , Clifford, C. , Haney, S. , Klein, M. K. , Beaver, L. , Vickery, K. , Vail, D. M. , Hershey, B. , Ettinger, S. , Vaughan, A. , Alvarez, F. , Hillman, L. , Kiselow, M. , Thamm, D. , Higginbotham, M. L. , Gauthier, M. , Krick, E. , … Gillings, S. (2012). Preliminary evidence for biologic activity of toceranib phosphate (Palladia(®)) in solid tumours. Veterinary and Comparative Oncology, 10(3), 194–205. 10.1111/j.1476-5829.2011.00275.x 22236194 PMC3732378

[vms370003-bib-0013] Marusyk, A. , Janiszewska, M. , & Polyak, K. (2020). Intratumor heterogeneity: The rosetta stone of therapy resistance. Cancer Cell, 37(4), 471–484. 10.1016/j.ccell.2020.03.007 32289271 PMC7181408

[vms370003-bib-0014] Miller, M. A. , Nelson, S. L. , Turk, J. R. , Pace, L. W. , Brown, T. P. , Shaw, D. P. , Fischer, J. R. , & Gosser, H. S. (1991). Cutaneous neoplasia in 340 cats. Veterinary Pathology, 28(5), 389–395. 10.1177/030098589102800506 1750164

[vms370003-bib-0015] Nakano, Y. , Kobayashi, M. , Bonkobara, M. , & Takanosu, M. (2017). Identification of a secondary mutation in the KIT kinase domain correlated with imatinib‐resistance in a canine mast cell tumor. Veterinary Immunology and Immunopathology, 188, 84–88. 10.1016/j.vetimm.2017.05.004 28615132

[vms370003-bib-0016] Nguyen, S. M. , Thamm, D. H. , Vail, D. M. , & London, C. A. (2015). Response evaluation criteria for solid tumours in dogs (v1.0): A veterinary cooperative oncology group (VCOG) consensus document. Veterinary and Comparative Oncology, 13(3), 176–183. 10.1111/vco.12032 23534501

[vms370003-bib-0017] Sabattini, S. , Guadagni Frizzon, M. , Gentilini, F. , Turba, M. E. , Capitani, O. , & Bettini, G. (2013). Prognostic significance of Kit receptor tyrosine kinase dysregulations in feline cutaneous mast cell tumours. Veterinary Pathology, 50(5), 797–805. 10.1177/0300985813476064 23377219

[vms370003-bib-0018] Serrano, C. , Mariño‐Enríquez, A. , Tao, D. L. , Ketzer, J. , Eilers, G. , Zhu, M. , Yu, C. , Mannan, A. M. , Rubin, B. P. , Demetri, G. D. , Raut, C. P. , Presnell, A. , McKinley, A. , Heinrich, M. C. , Czaplinski, J. T. , Sicinska, E. , Bauer, S. , George, S. , & Fletcher, J. A. (2019). Complementary activity of tyrosine kinase inhibitors against secondary kit mutations in imatinib‐resistant gastrointestinal stromal tumours. British Journal of Cancer, 120(6), 612–620. 10.1038/s41416-019-0389-6 30792533 PMC6462042

[vms370003-bib-0019] Tani, H. , Miyamoto, R. , Noguchi, S. , Kurita, S. , Nagashima, T. , Michishita, M. , Yayoshi, N. , Tamura, K. , & Bonkobara, M. (2021). A canine case of malignant melanoma carrying a KIT c.1725_1733del mutation treated with toceranib: A case report and in vitro analysis. BMC Veterinary Research, 17(1), 147. 10.1186/s12917-021-02864-3 33827546 PMC8028755

[vms370003-bib-0020] Thamm, D. H. , Weishaar, K. M. , Charles, J. B. , & Ehrhart, E. J., 3rd (2020). Phosphorylated KIT as a predictor of outcome in canine mast cell tumours treated with toceranib phosphate or vinblastine. Veterinary and Comparative Oncology, 18(2), 169–175. 10.1111/vco.12525 31365175

[vms370003-bib-0021] Webster, J. D. , Yuzbasiyan‐Gurkan, V. , Kaneene, J. B. , Miller, R. , Resau, J. H. , & Kiupel, M. (2006). The role of c‐KIT in tumorigenesis: Evaluation in canine cutaneous mast cell tumours. Neoplasia, 8(2), 104–111. 10.1593/neo.05622 16611403 PMC1578516

[vms370003-bib-0022] Yuzawa, S. , Opatowsky, Y. , Zhang, Z. , Mandiyan, V. , Lax, I. , & Schlessinger, J. (2007). Structural basis for activation of the receptor tyrosine kinase KIT by stem cell factor. Cell, 130(2), 323–334. 10.1016/j.cell.2007.05.055 17662946

