# Supplementary material for: A case report of feline mast cell tumour with intertumoral heterogeneity: Identification of secondary mutations c.998G>C and c.2383G>C in KIT after resistance to toceranib
Source: Vet Med Sci. 2024 Aug 23;10(5):e70003. doi: 10.1002/vms3.70003 (PMC11342349; doi:10.1002/vms3.70003)
Supplement: Supplementary file 1 — Supporting Information [file VMS3-10-e70003-s001.docx]

**Supplementary materials**

**Polymerase chain reaction (PCR) and direct-sequence analysis**

PCR analysis using CFX connect Real time system (Bio-Rad Laboratories Inc., California, USA) and TB Green^®^ Premix Ex Taq™ (Tli RNase H Plus) (TaKaRa-Bio, Shiga, Japan) and direct-sequence analysis was performed for feline *KIT* genomic exons 6, 8, 9, 11, 13, and 17. Genomic deoxyribonucleic acid was extracted from paraffin-embedded samples using the phenol-chloroform and ethanol precipitation methods. Intronic primer pairs were designed to amplify the entire *KIT* exonic regions, (Supplementary Table 1). The amplification products of genomic DNA were directly sequenced. The normal feline *KIT* complementary DNA nucleotide sequence (GenBank accession number NM_001009837.3) was employed as a reference for comparison to identify mutations and determine the numbering of nucleotide and amino acid sequence. used for comparison in order to determine the mutation and numbering of the nucleotide
