# Supplementary material for: A case report of feline mast cell tumour with intertumoral heterogeneity: Identification of secondary mutations c.998G>C and c.2383G>C in KIT after resistance to toceranib
Source: Vet Med Sci. 2024 Aug 23;10(5):e70003. doi: 10.1002/vms3.70003 (PMC11342349; doi:10.1002/vms3.70003)
Supplement: Supplementary file 2 — Supporting Information [file VMS3-10-e70003-s002.docx]

**Supplementary figure legend.**

Detection of secondary mutation of *KIT* exon 6 and 17. *KIT* c.998G>C in exon 6 (left panels) and *KIT* c.2393G>C in exon 17 (right panels) were identified using analysis of polymerase chain reaction-direct sequencing.
