# Supplementary figures and images for: A case report of feline mast cell tumour with intertumoral heterogeneity: Identification of secondary mutations c.998G>C and c.2383G>C in KIT after resistance to toceranib
Source: Vet Med Sci. 2024 Aug 23;10(5):e70003. doi: 10.1002/vms3.70003 (PMC11342349; doi:10.1002/vms3.70003)

## Slide 1
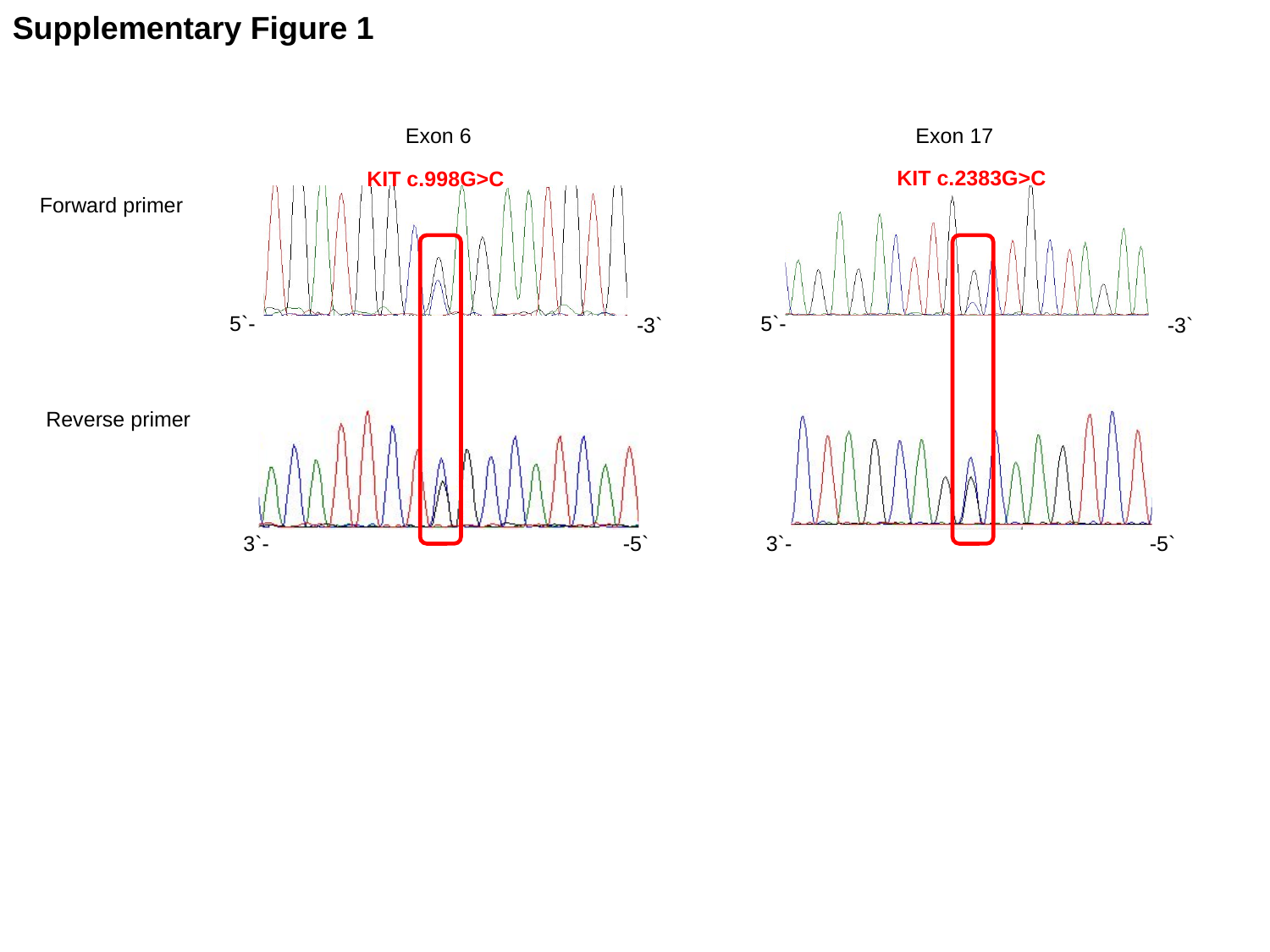

Supplementary Figure 1
Exon 6
Exon 17
KIT c.2383G>C
KIT c.998G>C
Forward primer
5`-
5`-
-3`
-3`
Reverse primer
3`-
-5`
3`-
-5`

Supplement: Supplementary file 3 — Supporting Information [file VMS3-10-e70003-s003.pptx]
