# Supplementary material for: A case report of feline mast cell tumour with intertumoral heterogeneity: Identification of secondary mutations c.998G>C and c.2383G>C in KIT after resistance to toceranib
Source: Vet Med Sci. 2024 Aug 23;10(5):e70003. doi: 10.1002/vms3.70003 (PMC11342349; doi:10.1002/vms3.70003)
Supplement: Supplementary file 4 — Supporting Information [file VMS3-10-e70003-s004.docx]

| **Supplementary table 1. Primer pairs for feline KIT.** | | |
| --- | --- | --- |
| Exon | Primer | Sequence (5` to 3`) |
| 6 | Forward | TTCCGTTGGATGGCTGTG |
|  | Reverse | GTAAGCCAAGGAGAGACAGG |
| 8 | Forward | TGAAAGCAAGGGAGGGAGGAAGTC |
|  | Reverse | GTCCTTCCCTTACGCATGTC |
| 9 | Forward | GATGGTTGATCTTTCTGGAG |
|  | Reverse | AATCATGACTGATATGGCAGGC |
| 11 | Forward | CTCCCCTAATAAGCGCTGTAATGA |
|  | Reverse | CAGGTGCAACAGAACAAAGGAAGT |
| 13 | Forward | ATTGGCTTGCCAGATGTA |
|  | Reverse | GCAAGAAGCTTATAATCGAGC |
| 17 | Forward | GTGTGACAGAAGCAGCATC |
|  | Reverse | GAGACTAACATCCTTCATTGG |
